# Serotype distribution and antibiotic resistance of *Streptococcus pneumoniae* isolates collected at a Chinese hospital from 2011 to 2013

**DOI:** 10.1186/s12879-015-1042-5

**Published:** 2015-08-05

**Authors:** Songyin Huang, Xiaoqiang Liu, Weisi Lao, Suhua Zeng, Huiqi Liang, Rihui Zhong, Xinlu Dai, Xiquan Wu, Hongyu Li, Yandan Yao

**Affiliations:** Department of Laboratory, Sun Yat-sen Memorial Hospital, Sun Yat-sen University, Guangzhou, Guangdong 510120 China; Guangdong Provincial Key Laboratory of Malignant Tumor Epigenetics and Gene Regulation, Sun Yat-sen Memorial Hospital, Sun Yat-sen University, Guangzhou, Guangdong 510120 China; Department of Ophthalmic Surgery, Zhongshan Ophthalmic Center, Sun Yat-sen University, Guangzhou, Guangdong 510060 China; Breast Tumor Center, Sun Yat-sen Memorial Hospital, Sun Yat-sen University, Guangzhou, Guangdong 510120 China; General Surgery, Sun Yat-sen Memorial Hospital, Sun Yat-sen University, Guangzhou, Guangdong 510120 China

**Keywords:** Multiple drug resistance, Serotype, Serogroup, *Streptococcus pneumoniae*, Community-acquired infections, Vaccines for indexing purposes

## Abstract

**Background:**

*Streptococcus pneumoniae* infections are a major cause of global morbidity and mortality, and the emergence of antibiotic-resistant *Streptococcus pneumoniae* strains has been increasingly reported. This study provides up-to-date information on bacterial serotype distribution and drug resistance from *S. pneumoniae* clinical isolates that could guide prevention and treatment strategies for pneumococcal disease in China.

**Methods:**

A total of 94 *S. pneumoniae* isolates were collected from outpatients and inpatients at one Chinese hospital from 2011–2013. Drug susceptibility and resistance was determined by minimum inhibitory concentrations (MICs). Capsular serotypes were identified by the quellung reaction test and multiplex polymerase chain reaction.

**Results:**

Fifteen serotypes were identified among the 94 *S. pneumoniae* clinical isolates that were collected. Prevalent serotypes were 19F (42.6 %), 19A (8.5 %), 3 (8.5 %), and 6B (7.4 %). Potential immunization coverage rates for the 7-, 10- and 13-valent pneumococcal polysaccharide conjugate vaccines were 59.6, 62.6, and 79.6 %, respectively. Resistance rates to tetracycline, erythromycin, and trimethoprim/sulfamethoxazole were 91.2, 80.2 and 63.8 %, respectively. Resistance rates to penicillin, amoxicillin, ceftriaxone, and cefotaxime were 47.3, 34.1, 19.8, and 18.7 %, respectively. In almost all cases, antimicrobial resistance of the *S. pneumoniae* isolates in patients five years or younger was higher than isolates collected from patients aged 51 years or older.

**Conclusion:**

Prevalent serotypes among the 94 *S. pneumoniae* clinical isolates were 19F, 19A, 3, and 6B. The 13-valent pneumococcal polysaccharide conjugate vaccine covered the majority of the serotypes identified in this sample. Drug resistance varied among different serotypes and age groups. Clinical precautions should be taken to avoid the development of multidrug resistance in this potential human pathogen.

**Electronic supplementary material:**

The online version of this article (doi:10.1186/s12879-015-1042-5) contains supplementary material, which is available to authorized users.

## Background

Gram-positive *Streptococcus pneumoniae* is a leading cause of global morbidity and mortality in children and adults, causing pneumonia, bacteremia, sepsis syndromes, otitis media (primarily in children), and meningitis [1]. The increasing incidence of antibiotic-resistant *S. pneumoniae* strains worldwide poses a threat to the effective treatment of these infections. The burden of pneumococcal diseases is worsened by increasing numbers of people affected with chronic disease (e.g. sickle-cell disease, chronic renal failure, chronic liver disease, asplenia, and human immunodeficiency virus) or mycobacterial infection, as well as an aging population in many developed countries [2, 3]. Approximately 70–100 million children aged under 5 years die of *S. pneumoniae* infection annually, and 90 % of these deaths occur in developing countries [4]. Data from China show that there were 12,815 cases/100,000/year of all-cause pneumonia and 526 deaths/100,000 among children aged 1 to 59 months, and there were 14 cases/100,000/year of meningitis between 1980 and 2008 [5]. Importantly, *S. pneumoniae* is the most dominant causative agent of community-acquired pneumonia among pediatric patients [5–7].

The antigenicity of the *S. pneumoniae* capsular polysaccharide is diverse, and infection or vaccination with *S. pneumoniae* can stimulate the production of a specific protective antibody that exhibits cross protection against certain serotypes. A number of multivalent vaccines have been developed to reduce the disease burden caused by specific pneumococcal serotypes. The recently developed polysaccharide conjugate vaccine (PPV) PPV23 vaccine contains purified capsular polysaccharides from 23 serotypes (1, 2, 3, 4, 5, 6B, 7F, 8, 9N, 9V, 10A, 11A, 12F, 14, 15B, 17F, 18C, 19A, 19F, 20, 22F, 23F and 33F) that collectively account for 85–90 % of invasive pneumococcal disease (IPD) cases among adults; however, it is not recommended for infants. The *S. pneumoniae* polysaccharide capsule largely determines virulence and this structure remains the target of many current pneumococcal vaccines. Notably, there are at least 94 *S. pneumoniae* serotypes that differ in their nasopharyngeal carriage prevalence, invasiveness, and disease incidence. Some of these serotypes exhibit a distinctive epidemiology that includes their potential to cause invasive disease, their incidence in specific age groups and geographic regions, and their resistance to different antibiotics [1].

Antibiotic-resistant pneumococci have been steadily increasing since the 1990s and are becoming a major problem worldwide. The emergence of multidrug-resistant *S. pneumoniae* (MDRSP) has been observed in various countries over the past several decades. The growing resistance of *S. pneumoniae* to commonly used antibiotics emphasizes the urgent need for effective vaccines that control pneumococcal disease. A subset of capsular polysaccharides cloned from clinically important serotypes is included in PCV. The introduction of PCVs into national immunization programs can substantially decrease the incidence of IPD caused by vaccine-type pneumococci in many countries [8–10]. More specifically, PPV PCV7, PCV10 and PCV13 have proven effective in decreasing the infection rate of *S. pneumoniae* and preventing the emergence of MDRSP [11, 12].

The aim of this study was to determine the serotype distribution and antimicrobial susceptibility of *S. pneumoniae* isolates among pediatric and older patients, as well as the immunization coverage rates for PCV7 (covering serotypes 4, 6B, 9V, 14, 18C, 19F and 23F), PCV10 (PCV7 plus serotypes 1, 5 and 7F) and PCV13 (PCV10 plus serotypes 3, 6A and 19A).

## Methods

### Clinical isolates and culture conditions

This study was conducted at the Sun Yat-Sen Memorial Hospital, a teaching hospital located in Guangzhou, China. A total of 94 pneumococcal isolates collected at the hospital from January 2011 to June 2013 were included in this study. The study was approved by the Ethics Committee of Sun Yat-Sen Memorial Hospital and each patient or parent guardian from which the isolates were collected provided informed consent prior to study commencement.

Invasive *S. pneumoniae* isolates were recovered from clinical specimens of normally sterile body sites such as blood (10.6 %), cerebral spinal fluid (CSF) (6.4 %), and pleural fluid (7.4 %). Non-invasive *S. pneumoniae* isolates were recovered from clinical specimens such as sputum, tracheal/bronchial aspirates (lower respiratory tract specimens) (60.6 %), and ear secretions (14.9 %). To avoid sample duplication, isolates that were consecutively isolated from the same individual were excluded. For each patient, data on demographics and clinical, radiological, and other outcomes relevant to the study were recorded and analyzed. Patients’ ages ranged between 1 month and 92 years, with an average of 40 ± 24 years. The male to female ratio was 2.07: 1 (no. of males = 64, 68 %; no. of females = 30, 32 %).

Gram-positive samples were grown on 5 % sheep blood agar and incubated at 37 °C in the presence of 5 % CO_2_ for 18–24 h prior to biochemical and molecular assays. All isolates were stored at −80 °C in a fat-free milk preservation medium until analyses were performed.

### Identification of isolates

*S. pneumoniae* isolates were identified by colony morphology, the alpha hemolysis test, the optochin sensitivity test (Oxoid, Basingstoke, UK), bile solubility, the catalase test, and the Omni antiserum assay (Statens Serum Institute, Copenhagen, Denmark). Conventional microbiological methods were used in combination with the VITEK® 2 microbial analysis system according to the manufacturer’s instructions (bioMérieux, La Balme-les-Grottes, France).

### Susceptibility testing

The antimicrobial susceptibility of each isolate was tested using the VITEK® 2 *S. pneumoniae* susceptibility card (AST-GP68, bioMérieux) according to the 2013 guidelines created by the Clinical and Laboratory Standards Institute (CLSI). >The minimum inhibitory concentration (MIC) of each isolate was determined against the following 15 antimicrobial agents: penicillin, amoxicillin, cefotaxime, ceftriaxone, ertapenem, meropenem, erythromycin, levofloxacin, moxifloxacin, ofloxacin, linezolid, vancomycin, tetracycline, chloramphenicol, and trimethoprim/sulfamethoxazole. For penicillin, meningitis criteria were set as follows: susceptible, MIC ≤ 0.06 μg/mL; resistant, MIC ≥ 0.12 μg/mL. Non-meningitis criteria for penicillin were as follows: susceptible, MIC ≤ 2 μg/mL; resistant, MIC ≥ 8 μg/mL. For cefotaxime and ceftriaxone, meningitis criteria were as follows: susceptible, MIC ≤ 0.5 μg/mL; resistant, MIC ≥ 2 μg/mL. Non-meningitis criteria for cefotaxime and ceftriaxone were set as follows: susceptible, MIC ≤ 1 μg/mL; resistant, MIC ≥ 4 μg/ml. Isolate ATCC 49619 and *Staphylococcus aureus* ATCC 29213 were used as quality control strains during susceptibility testing. MDRSP isolates were defined as isolates that were resistant to three or more classes of antimicrobial agents.

### Serotyping

All isolates were serotyped by a capsular quellung reaction test using type-specific antisera (Statens Serum Institute, Copenhagen, Denmark) against serotypes present in the 23-valent PPV (i.e. 1, 2, 3, 4, 5, 6B, 7F, 8, 9N, 9V, 10A, 11A, 12F, 14, 15B, 17F, 18C, 19A, 19F, 20, 22F, 23F, and 33F). Serotyping was performed by phase-contrast microscopy as described previously [13]. Strains that could not be serotyped by a quellung reaction test were serotyped using multiplex PCR [14].

### Molecular capsular typing

Genomic DNA was extracted from the bacterial cultures using the DNeasy Blood & Tissue Kit (Qiagen Multiplex PCR Kit, Hilden, Germany). Primer sequences were published by the Center for Disease Control and Prevention (CDC) (http://www.cdc.gov/streplab/pcr.html). A total of 28 primers were grouped into nine multiplex reactions. Primers used in this study are listed in Additional file 1: Table S1 and PCR reactions conditions were performed as described previously [15], with minor modifications. Briefly, multiplex PCR was performed in 50 μl volumes and each reaction mixture contained the following reagents: 5 μl 10 × PCR buffer (Qiagen Multiplex PCR Kit), 5 μl DNA sample, 1 μl dNTPs (50 μM/μl), 1 μl Taq (5 U/μl), 37 μl H_2_O; 0.5 μl Primer F; and 0.5 μl Primer R. PCR tubes were placed in a thermocycler (DNA Engine, BioRad, Hercules, California, USA) programmed for initial denaturation at 94 °C × 4 min, followed by 30 amplification cycles at 94 °C × 45 s, annealing at 54 °C × 45 s, extension at 72 °C × 2 min, and a final extension at 72 °C × 10 min. PCR products were electrophoresed in a 2 % agarose gel at 50–100 volts for 40 min, stained with ethidium bromide, and visualized by transillumination (Ultra-Violet Products Ltd., Upland, California, USA).

### Statistical analysis

All data were analyzed with WHONET software (version 5.6, WHO Collaborating Centre). Statistical comparisons were made using the Chi-squared and Fisher’s exact tests calculated by SPSS software (version 19.0). Two-tailed *P* values < 0.05 were considered statistically significant.

## Results and discussion

### Demographics

**Fig. 1:**
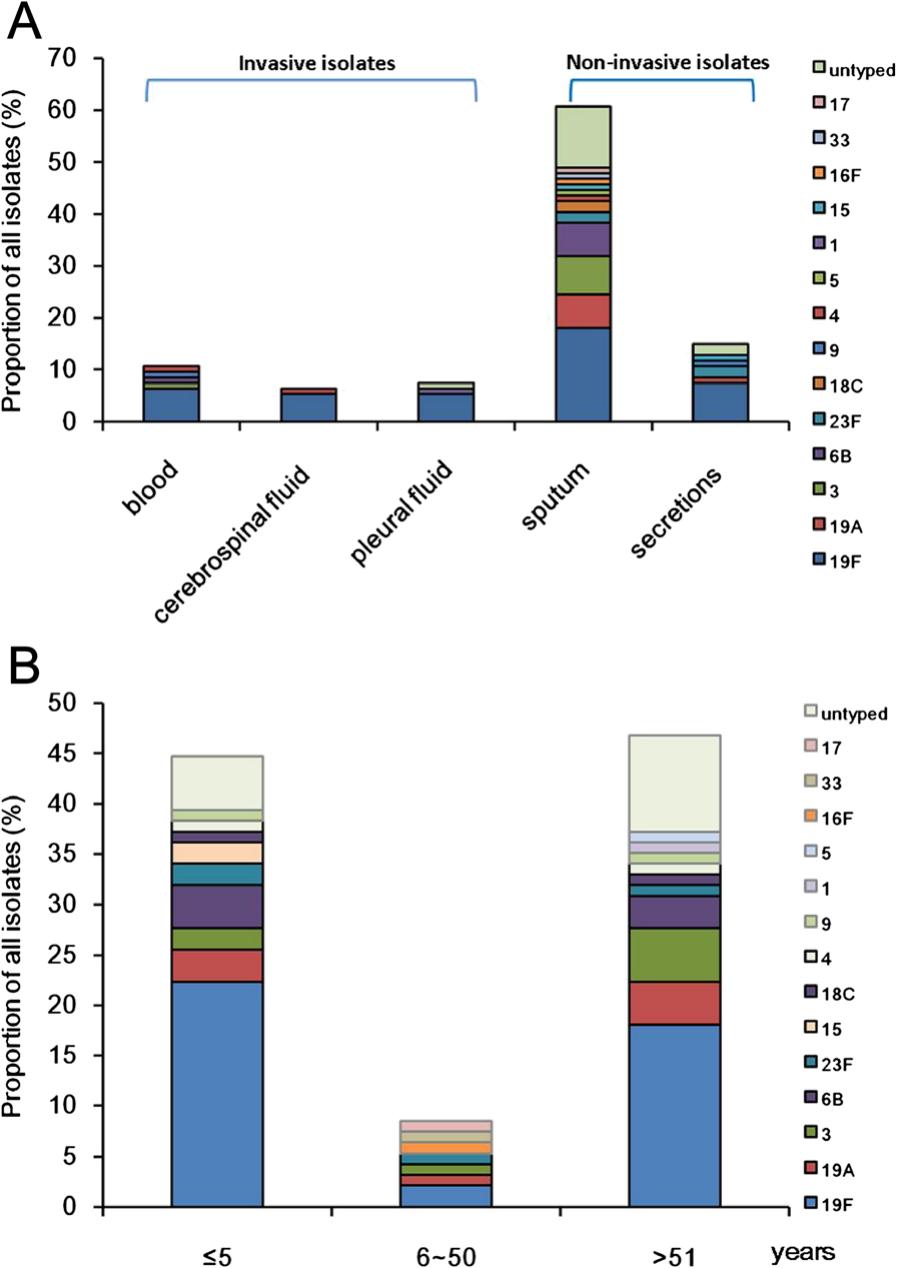
Serotype distribution among clinical specimens and age groups. **a**. Proportion and serotype distribution of *S. pneumoniae* isolates collected from different specimen sources. **b**. Proportion and serotype distribution of *S. pneumoniae* isolates among the different age groups

A total of 94 *S. pneumoniae* isolates were prospectively collected from patients with pneumococcal infections at one Chinese hospital from 2011 to 2013. Of these, the most prevalent specimen sources were sputum and tracheal/bronchial aspirates (lower respiratory tract specimens) (*n* = 57, 60.6 %), followed by ear secretions (*n* = 14, 14.9 %), blood (*n* = 10, 10.6 %), CSF (*n* = 6, 6.4 %), and pleural fluid (*n* = 7, 7.4 %) (Fig. 1a, Additional file 1: Table S2). The rate of pneumococcal infections was higher in male patients (68 %) than in female patients (32 %), but this difference was not statistically significant (*P* = 0.61). Chaïbou et al. reported similar infection rates among males in Burkina Faso (257/476, 55 %) and findings by Bos et al. were similar among males in Mozambique (150/177, 85 %) [16, 17]. In contrast to patient sex, the serotype distribution displayed some age-dependent variation (Fig. 1b, Additional file 1: Table S3). Serotype proportions were significantly different between all three defined age groups (≤5 years, 6 ~ 50 years, and ≥ 51 years), but these differences may have been related to the limited number of serotypes present. Among the 94 isolates, 42 (44.7 %) were isolated from children (≤5 years) and 44 (46.8 %) were isolated from patients ≥ 51 years.

### Serotype distribution and immunization coverage

**Fig. 2:**
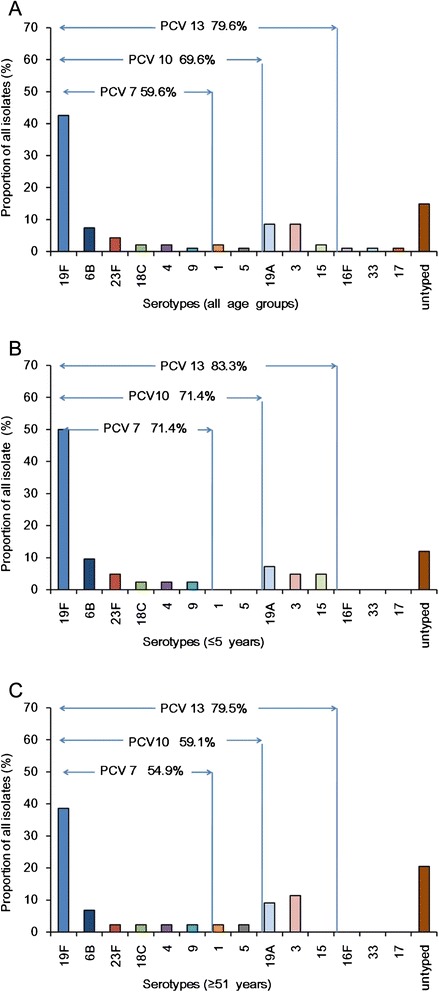
Serotype distribution and PCV immunization coverage among the *S. pneumoniae* isolates. **a**. Serotypes and immunization coverage rates of PCVs among all age groups. **b**. Serotypes and immunization coverage rates of PCVs among children ≤ 5 years. **c**. Serotypes and immunization coverage rates of PCVs among patients ≤ 51 years

Of the 94 *S. pneumoniae* isolates analyzed, 54 were identifiable by capsular serotyping. Of the remaining 40 isolates, 26 were identified using multiplex PCR, and 14 isolates (14.9 %) could not be serotyped. The serotype distribution of these *S. pneumoniae* isolates is shown in Fig. 2a and Additional file 1: Table S4. The most commonly isolated serotypes were 19F (*n* = 40, 42.6 %), 19A (*n* = 8, 8.5 %), 3 (*n* = 8, 8.5 %), 6B (*n* = 7, 7.4 %), and 23F (*n* = 4, 4.3 %). These five serotypes accounted for 71.3 % of the isolates. Serotypes 19F and 19A were the two dominant types. Interestingly, we did not find any recently discovered serotype 6C and 6D strains that were reportedly isolated from [18, 19].

Serotype distributions varied according to age (Fig. 1b, 2b, c and Additional file 1: Table S3). Serotype distribution was similar in patients ≤ 5 years and patients older than 51 years. However, the rank order of these serotypes varied. Among the patients who were younger than 5 years, the most common serotypes were 19F (50 % of isolates), 6B (9.5 %), 19A (7.1 %), 3 (4.8 %), and 15 (4.8 %); these accounted for 73.8 % of the isolates in this age group. In contrast, the most common serotypes in patients 51 years or older were 19F (38.6 %), 3 (11.4 %), 19A (9.1 %), 6B (6.8 %), and 23F (2.3 %); these accounted for 68.2 % of the isolates in this age group. Serotypes 16F, 17, and 33 were only detected in patients aged between 6 and 50 years (Fig. 1b, Additional file 1: Table S3).

PPVs comprise a combination of pneumococcal polysaccharide with an immunogenic protein carrier that can enhance the host’s antibody response and induce immunological memory. PCV7 covered serotypes 4 6B, 9V, 14, 18C, 19F, and 23F. PCV10 covered all serotypes covered by PCV7, as well as serotypes 1, 5, and 7F. PCV13 covered all serotypes covered by PCV10, as well as serotypes 3, 6A, and 19A. Overall immunization coverage rates for PCV13, PCV10, and PCV7 were 79.6, 62.6, and 59.6 %, respectively. Importantly, we determined that PCV immunization coverage is higher in children younger than 5 years, despite numerous reports that this age group is at the highest risk of developing IPD [7]. However, there was no significant difference in the proportions of PCV7-specific serotypes, PCV10-specific serotypes, or PCV13-specific serotypes of patients younger than 5 years (71.4, 71.4, and 83.3 %, respectively) and those patients 51 years or older (54.9, 59.1, and 79.5 %, respectively). Furthermore, the proportion of PCV10-specific serotypes was lower in patients younger than 5 years than in patients 51 years or older (0 versus 4.4 %, *P* = 0.23). Last, the proportion of PCV13-specific serotypes was similarly lower in patients younger than 5 years (11.9 % versus 20.0 %, *P* = 0.03) (Fig. 2b, c and Additional file 1: Table S5).

### Antimicrobial susceptibility

**Fig. 3:**
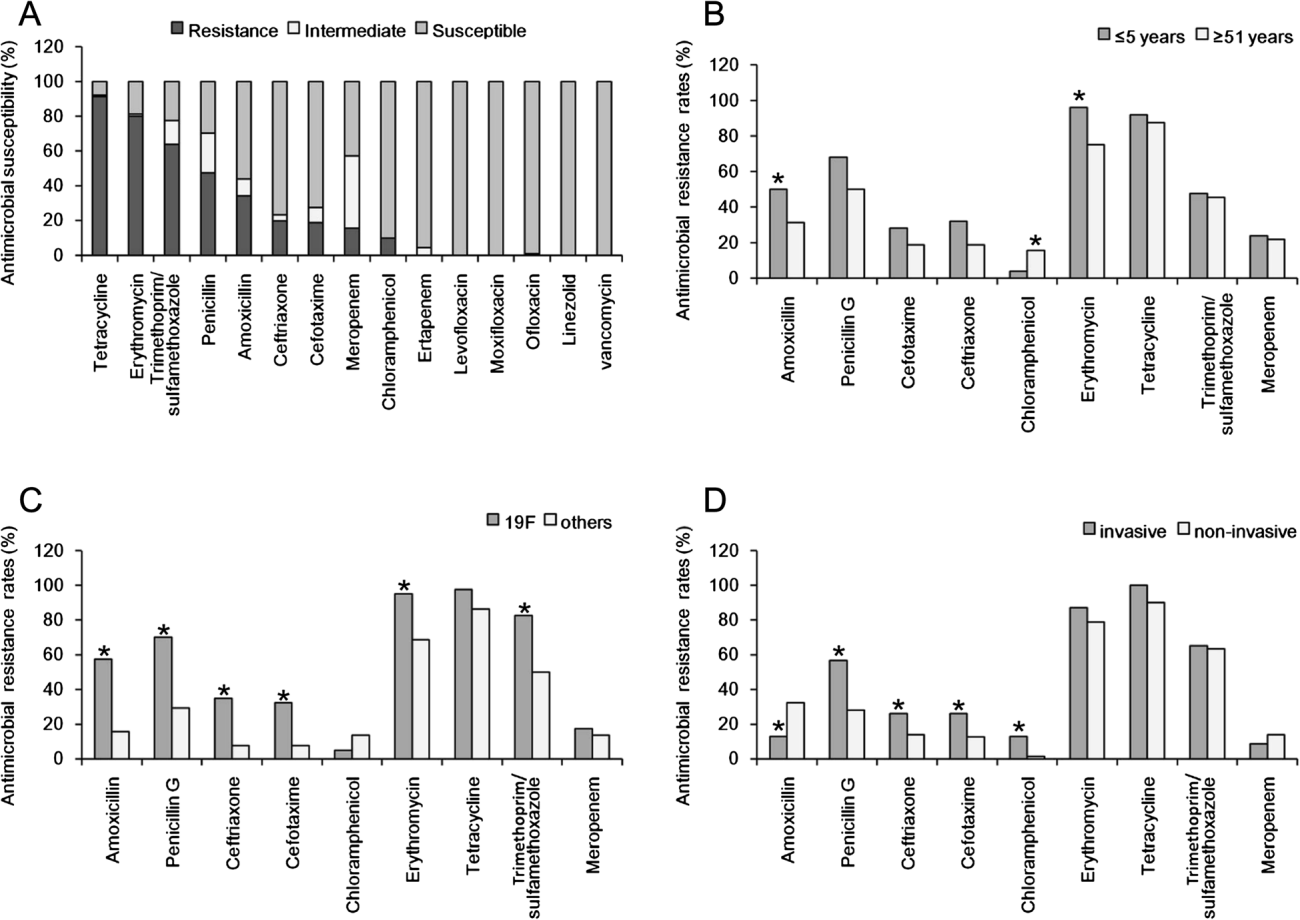
Antibiotic susceptibility of *S. pneumoniae* isolates. **a**. Antibiotic susceptibility. **b**. Comparison of antibiotic resistance between patients ≤ 5 years and patients ≤ 51 years. **c**. Comparison of antibiotic resistance between serotype 19F and other serotypes. **d**. Comparison of antibiotic resistance between invasive and non-invasive *S. pneumoniae* isolates. * *P* < 0.05

Antimicrobial susceptibilities determined for the clinical isolates analyzed in this study are shown in Fig. 3a and Additional file 1: Table S6. The percentage of these isolates that conferred resistance to tetracycline, erythromycin, and trimethoprim/sulfamethoxazole was 91.2, 80.2, and 63.8 %, respectively; the percentage of isolates that conferred resistance to penicillin and amoxicillin was 47.3 and 34.1 %, respectively; and the percentage of isolates that conferred resistance to ceftriaxone and cefotaxime was 19.8 and 18.7 %, respectively. All isolates were susceptible to ertapenem, quinolone, linezolid and vancomycin, with the exception of one isolate (1.1 %) that was intermediately susceptible to ofloxacin, and 4 (4.4 %) isolates that were intermediately susceptible to ertapenem.

Almost all antimicrobial resistance rates for isolates collected from the pediatric patient group (≤5 years) were higher than those collected from patients 51 years or older (Table 1). More specifically, the percentage of *S. pneumoniae* isolates that was resistant to amoxicillin and erythromycin was significantly higher in the pediatric group than in patients aged 51 years or older (*P* < 0.05) (Fig. 3b). Because 19F was the dominant serotype, we also compared the antimicrobial susceptibility of 19 F to that of the other types. Serotype 19F had a higher rate of resistance to penicillin, amoxicillin, ceftriaxone, cefotaxime, erythromycin, and trimethoprim/sulfamethoxazole than all other serotypes (Fig. 3c and Additional file 1: Table S6; *P* < 0.05).

According to the revised CLSI breakpoints for parenteral penicillin (i.e. MIC ≥ 8 μg/mL for resistant non-meningitis isolates and MIC ≥ 0.12 μg/mL for resistant meningitis isolates), the proportion of invasive and non-invasive isolates that conferred penicillin resistance was 56.5 and 28.2 %, respectively. The proportion of invasive and non-invasive isolates that conferred resistance to cefotaxime was 26.1 and 12.7 %, respectively, and the proportion of invasive and non-invasive isolates that conferred resistance to ceftriaxone was 26.1 and 14.1 %, respectively. In summary, these results indicate that the resistance rates of these *S. pneumoniae* isolates to multiple antibiotics, with the exception of meropenem and amoxicillin, were much higher in invasive infections than in non-invasive infections.

A MDR phenotype (resistance to ≥ 3 antibiotic classes) was found in 73.4 % (*n* = 69) of these isolates (61.7 and 6.4 % in the non-meningitis and meningitis isolates, respectively). The most common MDR pattern observed was resistance to erythromycin, tetracycline, and trimethoprim/sulfamethoxazole (*n* = 58, 61.7 %), followed by resistance to penicillin antibiotics, erythromycin, and tetracycline (*n* = 49, 52.1 %). Notably, antibiotic resistance was limited to a number of specific pneumococcal serotypes. A high MDR rate was primarily found in serotypes 19F (*n* = 37/94, 39.4 %) and 19A (*n* = 7/94, 7.4 %) serotypes. However, PCV immunization coverage included the majority of the serotypes that conferred MDR phenotypes (PCV7, 71.0 %; PCV10, 72.5 %; and PCV13, 89.9 %). In addition, most penicillin-nonsusceptible isolates were covered by PCVs (Table 2).

**Table 1:** Susceptibility of 15 antimicrobials for *S. pneumoniae* isolates from different groups

Antibiotics	Resistance of age groups (%)			Resistance of serotypes (%)			Resistance of specimens (%)		
	≤5 years (*n* = 42)	≥51 years (*n* = 44)	*p*-value	19F (*n* = 40)	Others (*n* = 54)	*p*-value	Invasive (*n* = 23)	Non-invasive (*n* = 71)	*p*-value
Amoxicillin	50.0	31.2	0.04	57.5	15.7	0.04	13.0	32.4	0.04
Penicillin G	68.0	50.0	0.28	70.0	29.4	0.04	56.5	28.2	0.03
Cefotaxime	28.0	18.8	0.12	32.5	7.8	0.05	26.1	12.7	0.04
Ceftriaxone	32.0	18.8	0.11	35.0	7.8	0.02	26.1	14.1	0.04
Chloramphenicol	4.0	15.6	0.04	5.0	13.7	0.16	13.0	1.4	0.01
Erythromycin	96.0	75.0	0.04	95.0	68.6	0.03	87.0	78.9	0.35
Tetracycline	92.0	87.5	0.41	97.5	86.3	0.39	100	90.1	0.23
Trimethoprim/sulfamethoxazole	47.6	45.5	0.22	82.5	50.0	0.02	65.2	63.4	0.29
Meropenem	24.0	21.9	0.27	17.5	13.7	0.51	8.7	14.1	0.03
Ertapenem	0	0	-	0	0	-	0	0	-
Levofloxacin	0	0	-	0	0	-	0	0	-
Moxifloxacin	0	0	-	0	0	-	0	0	-
Ofloxacin	0	0	-	0	0	-	0	0	-
Linezolid	0	0	-	0	0	-	0	0	-
Vancomycin	0	0	-	0	0	-	0	0	-

**Table 2:** Antimicrobial resistance of *S. pneumoniae* isolates

The serotypes of isolates	% of resistant isolates							
	Penicillins	Erythromycin	Tetracycline	Trimethoprim/sulfamethoxazole	Cephalosporin	Chloramphenicol	Meropenem	MDR^b,c^
All serotypes (*n* = 94)	47.3	80.2	91.2	63.8	19.8	9.9	15.4	73.4
Serotype								
19F (*n* = 40)	27.9	40.4	41.5	35.1	14.1	3.4	9.7	39.4
19A (*n*= 8)	4.3	8.5	8.5	7.4	0	0	0	7.4
6B (*n* = 7)	3.2	6.4	6.4	2.1	0	0	0	4.3
3 (*n* = 8)	2.1	2.1	7.4	2.1	1.1	0	0	2.1
Otherserotypes^a^								
(*n* = 18)	4.9	13.2	14.6	10.7	3.5	5.3	3.4	12.8
Untyped (*n* = 14)	4.9	9.6	12.8	6.4	1.1	1.2	2.3	7.4

^a^Included are 11 serotypes

^b^Multidrug-resistant isolates, i.e. resistant to three or four tested antimicrobials

^c^The PCVs covered the majority of serotypes that had MDR (*n* = 69) phenotypes [PCV7, 71.0 % (49/69); PCV10, 72.5 % (50/69); and PCV13, 89.9 % (62/69)]

*S. pneumoniae* normally inhabits the nasopharynx, throat, and oral cavity. However, pneumococcal infections remain a major medical problem as they are a major cause of morbidity and mortality in children and adults, and emerging resistance to conventional antibiotics further complicates this issue. Tracking serotype distribution and antibiotic resistance patterns may prove useful in the development and effective use of PCVs.

To date, *S. pneumoniae* serotype distribution reportedly varies by age, geography, disease, and time [1, 20]. One study of 2173 *S. pneumoniae* isolates collected worldwide between 2004 and 2009 showed that the most common serotypes found in Latin America were serotypes 1, 6A, and 14. In South Africa/Mauritius, the most common serotypes were 19F, 6B, 4 and 23F, while, serotype 19A was the most common among North American isolate and it was more prevalent among children than adults [1]. In Europe, the most commonly isolated *S. pneumoniae* serotypes were 3,,14, 1 and 7F [1]. In Shenzhen, China, the most common serotypes of invasive *S. pneumoniae* isolates were found children 14 years or younger; these included serotypes 19F, 14, 23F, 19A and 6B [7, 21]. In this study, we determined that the most common serotype in clinical isolates sampled at a hospital in Guangzhou, China is 19F, and this is in agreement with findings from other recent studies from China, Malaysia, and other Southeast Asian countries [19–21]. Other common serotypes that we identified in our study (19A, 6B, and 23F, are in agreement with previous reports as well [21–23]. However, in contrast to prior studies [21–24], we found that serotypes 3 and 15 were also common serotypes in our sample. These data suggest that serotype distribution differs regionally.

Age is a risk factor for pneumococcal infections [5, 16, 20, 25]. The age profile of the patients infected with *S. pneumoniae* in this study exhibits a typical twin-peak distribution. Most patients in our cohort were infected with serotypes 19F, 6B, or 19A, and serotype distribution among pediatric patients and patients 51 years or older were consistent with the overall serotype distribution.

As *S. pneumoniae* spreads in a community, serotype distribution among one age group can directly and indirectly affect serotype distribution in other age groups depending on community mixing patterns [26]. In one study, a decrease in PCV7 serotypes among children younger than 5 years was observed over a 1-year period, and a similar decrease in PCV7 serotypes was also observed in adults 65 years or older [27]. PCV7 was the first approved *S. pneumoniae* conjugate vaccine, and it has been put to use in more than 70 countries [11, 28]. In many industrialized countries, the widespread use of this vaccine has led to a dramatic decline in PCV7-serotype IPDs not only in vaccinated children, but also in the general unvaccinated population [29–31]. This may have partially contributed to a shift toward non-PCV7 serotype IPDs, and the observed increase in serotype 19A. It is also worth noting that the antimicrobial resistance of *S. pneumoniae* serotype 19A is evolving with the widespread use of antibiotics [32–34].

PCV immunization coverage varies among different populations and geographic regions [35]. Reports suggest that after switching from PCV7 to PCV10, the proportion of serotypes covered has increased to varying degrees in the USA, Europe, Africa and Asia, and switching from PCV10 to PCV13 further improved global immunization coverage by 4–7 % [12, 36]. Among children with pneumonia in five Chinese hospitals, replacing PCV7 with PCV10 increased immunization coverage rates by approximately 0.6 %, whereas replacing PCV10 with PCV13 increased coverage by approximately 15.4 % [37]. We found that the overall immunization coverage rates of PCV7, PCV10, and PCV13 were 59.6, 62.6 and 79.6 %, respectively, and these findings are similar to those reported by Ma [7]. However, the immunization coverage rates for these vaccines were higher in pediatric patients than in patients 51 years or older, although this difference was not significant. Immunization coverage for serotypes 3, 15, and 19A by PCV13 may be responsible for the significant increase in coverage observed when switching from PCV7 and PCV10 to PCV13. We suggest that PCV13 be introduced into the vaccination program in Guangzhou, China as it may protect a greater proportion of the Chinese population from developing pneumococcal disease.

Over the past two decades, the incidence of pneumococcal infections caused by antimicrobial-resistant pneumococci has increased [38]. To date, the epidemiology of *S. pneumoniae* and potential risk factors for the development of MDR have not been assessed in China. This knowledge is essential for selecting the most appropriate empirical antimicrobial therapy for these infections. Recently, the Asian Network for Surveillance of Resistant Pathogens (ANSORP) study group performed a prospective surveillance study on serious pneumococcal infections in Asian countries. Antibiotic resistance to β-lactams and macrolides was commonly observed. Furthermore, high prevalence rates (≥60 %) of penicillin-nonsusceptible *S. pneumoniae* (PNSP) were reported for Taiwan, Korea, Japan, and Vietnam, while Europe and the US had prevalence rates of approximately 30.0 % [38]. In China, increasing cross-resistance to penicillin and erythromycin has been observed, with a higher pneumococcal resistance rate to erythromycin than in the western countries [5, 39]. In addition, the reported infection rate of PNSP among children in Guangzhou, China was 78.0 % from 2001 to 2002, and 83.6 % in 2008 [15]. Of 171 invasive *S. pneumoniae* isolates sampled from 11 hospitals in 11 cities from 2006–2008, 96.0 and 75.0 % of isolates conferred resistance to erythromycin and trimethoprim/sulfamethoxazole, respectively, while 77.0 and 30.0 % of meningeal isolates conferred resistance to penicillin and ceftriaxone, respectively [21]. In this study, we determined that the PNSP prevalence rate was 70.4 %, while resistance rates to erythromycin and tetracycline were 80.2 and 91.2 %, respectively. These data demonstrate that penicillin, erythromycin, and tetracycline are not appropriate for treating *S. pneumoniae* infections in the Guangzhou region. Conversely, we found that resistance rates to ceftriaxone (19.8 %), cefotaxime (18.7 %), meropenem (15.4 %) are lower than those reported in other Chinese studies [7, 40]. This may be caused by different treatment strategies, specimen sources, or diseases studied in the Guangzhou area compared with other regions in China.

Levofloxacin is a broad-spectrum fluoroquinolone with greater antimicrobial activity against *S. pneumoniae* than older fluoroquinolones. However, increased use of fluoroquinolones has allowed for increasing emergence of levofloxacin-resistant pneumococci [41, 42]. Kang et al. [43] recently reported that approximately 4.7 % of pneumococcal isolates collected from individuals affected with community-acquired pneumonia in Asian countries were levofloxacin-nonsusceptible, and that independent risk factors for levofloxacin-nonsusceptible pneumococcal pneumonia were prior treatment with fluoroquinolones, cerebrovascular disease, and healthcare-associated infections. The dissemination of non-antimicrobial-susceptible clones has been a major factor in the emergence of antibiotic-resistant *S. pneumoniae* and pneumococcal resistance to fluoroquinolones is primarily caused by mutations in the quinolone resistance-determining regions of the *parC* and *gyrA* genes [44]. In the present study, we did not find any strains that were resistant to fluoroquinolones, but approximately 1.1 % of the isolates were ofloxacin-nonsusceptible. Although the prevalence of fluoroquinolone resistance at present remains low in this region, ongoing surveillance is necessary.

None of the isolates investigated in this work were resistant to ertapenem, linezolid or vancomycin. These data suggest that these drugs could replace penicillin as the first line of treatment for *S. pneumoniae* infections. However, it is important to note that serotype 19F is the most common type in China [5], and our results are in agreement that 19F had a higher rate of resistance to penicillin, ceftriaxone, cefotaxime, amoxicillin, and erythromycin. Moreover, 73.4 % of the *S. pneumoniae* isolates collected were MDRSP, and these results echo those of a previous study in Asian countries [38].

Together, these data confirm that antibiotic resistance is a serious problem in China. The inappropriate use of antibiotics may partially explain the high resistance rate observed, and MDR serotypes likely play an important role in this phenomenon. Fortunately, PCV7 and PCV13 cover 71.0–89.9 % of the MDR pneumococcal isolates collected. Thus, they both have potential for controlling MDRSP. Given that serotype 19A plays an important role in the development of *S. pneumoniae*-related infections in China, however, PCV13 may be more useful than PCV7.

## Conclusion

A higher incidence of *S. pneumoniae* infections exists among children five years or younger and in adults 51 years or older. Given the high prevalence and clinical impact of pneumococcal antibiotic resistance, continuous surveillance of pneumococcal epidemiology is strongly warranted. This study also provides important data on *S. pneumoniae* serotype distribution and immunization coverage that could influence vaccination strategies in Guangzhou, China. Importantly, drug resistance trends vary with different *S. pneumoniae* serotypes and patient age groups. Clinical precautions should be taken to avoid the emergence of MDR in *S. pneumoniae*. Results obtained from this study may provide clinical guidance for the use of antimicrobial agents and the scientific basis for the independent development of vaccine policy in Guangzhou, China.

## Additional file

Additional file 1: Table S1. List of primers used for *S. pneumonia* serotype deduction (http://www.cdc.gov/streplab/pcr.html). **Table S2.** Distribution of serotypes in different specimens. **Table S3.** Distribution of different serotypes in different age groups. **Table S4.** The serotypes distribution and coverage rates of vaccines of the 94 isolates of *S.pneumoniae.* **Table S5.** The serotype distribution and coverage rates of vaccines of *S.pneumoniae* isolates from children and elderly patients. **Table S6.** The antibiotic susceptibility of 94 strains of *S.pneumoniae.* (PDF 140 kb)

## Abbreviations

CSF: Cerebrospinal fluid
IPD: Invasive pneumococcal disease
MDR: Multidrug resistance
MDRSP: Multidrug-resistant *Streptococcus pneumoniae*
*S. pneumonia*: *Streptococcus pneumonia*
MIC: Minimum inhibitory concentration
PCR: Polymerase chain reaction
PCV: Pneumococcal polysaccharide conjugate vaccine
PNSP: Penicillin-nonsusceptible *Streptococcus pneumoniae*
