# Supplementary material for: Serotype distribution and antibiotic resistance of Streptococcus pneumoniae isolates collected at a Chinese hospital from 2011 to 2013
Source: BMC Infect Dis. 2015 Aug 5;15:312. doi: 10.1186/s12879-015-1042-5 (PMC4526307; doi:10.1186/s12879-015-1042-5)
Supplement: Additional file 1: Table S1. — List of primers used for S. pneumonia serotype deduction (http://www.cdc.gov/streplab/pcr.html). Table S2. Distribution of serotypes in different specimens. Table S3. Distribution of different serotypes in different age groups. Table S4. The serotypes distribution and coverage rates of vaccines of the 94 isolates of S.pneumoniae. Table S5. The serotype distribution and coverage rates of vaccines of S.pneumoniae isolates from children and elderly patients. Table S6. The antibiotic susceptibility of 94 strains of S.pneumoniae. (PDF 140 kb) [file 12879_2015_1042_MOESM1_ESM.pdf]

Supporting information

Table S1. List of primers used for *S. pneumonia* serotype deduction (http://www.cdc.gov/ncidod/biotech/pcr.htm)

Table S2. Distribution of serotypes in different specimens [% (n)]

Table S3. Distribution of different serotypes in different age groups (n)

Table S4. The serotypes distribution and coverage rates of vaccines of the 94 isolates of *S. pneumoniae*

Table S5. The serotype distribution and coverage rates of vaccines of *S.pneumoniae* isolates from children and elderly patients [%(n)]

Table S6. The antibiotic susceptibility of 94 strains of *S.pneumoniae* (%)

Table S1. List of primers used for *S. pneumonia* serotype deduction (http://www.cdc.gov/ncidod/biotech/pcr.htm).

Primers	Primer sequence（5'-3'）	Products size（bp）	concentration（μM）
1-f	CTC TAT AGA ATG GAG TAT ATA AAC TAT GGT TA	280	0.3
1-r	CCA AAG AAA ATA CTA ACA TTA TCA CAA TAT TGG C		0.3
3-f	ATG GTG TGA TTT CTC CTA GAT TGG AAA GTA G	371	0.3
3-r	CTT CTC CAA TTG CTT ACC AAG TGC AAT AAC G		0.3
4-f [a]	CTG TTA CTT GTT CTG GAC TCT CGA TAA TTG G	430	0.3
4-r	GCC CAC TCC TGT TAA AAT CCT ACC CGC ATT G		0.3
5-f	ATA CCT ACA CAA CTT CTG ATT ATG CCT TTG TG	362	0.3
5-r	GCT CGA TAA ACA TAA TCA ATA TTT GAA AAA GTA TG		0.3
6A/6B/6C/6D -f	AAT TTG TAT TTT ATT CAT GCC TAT ATC TGG	250	0.3
6A/6B/6C/6D -r	TTA GCG GAG ATA ATT TAA AAT GAT GAC TA		0.3
7F/7A-f	TCC AAA CTA TTA CAG TGG GAA TTA CGG	599	0.4
7F/7A-r	ATA GGA ATT GAG ATT GCC AAA GCG AC		0.4
9V/9A-f	GGG TTC AAA G TC AGA CAG TG A ATC TTA A	816	0.5
9V/9A-r	CCA TGA ATG A AA TCA ACA TT G TCA GTA GC		0.5
14-f	GAA ATG TTA CTT GGC GCA GGT GTC AGA ATT	189	0.3
14-r	GCC AAT ACT TCT TAG TCT CTC AGA TGA AT		0.3
15B/15C-f	TTG GAA TTT TTT AAT TAG TGG CTT ACC TA	496	0.3
15B/15C-r	CAT CCG CTT ATT AAT TGA AGT AAT CTG AAC C		0.3
18/(18A/18B/18C/18F)-f	CTT AAT AGC TCT CAT TAT TCT TTT TTT AAG CC	573	0.3
18/(18A/18B/18C/18F)-r	TTA TCT GTA AAC CAT ATC AGC ATC TGA AAC		0.3
19A-f	GAG AGA TTC ATA ATC TTG CAC TTA GCC A	566	0.3
19A-r	CAT AAT AGC TAC AAA TGA CTC ATC GCC		0.3
19F-f	GTT AAG ATT GCT GAT CGA TTA ATT GAT ATC C	304	0.5
19F-r	GTA ATA TGT CTT TAG GGC GTT TAT GGC GAT AG		0.5
23F-f [a]	GTA ACA GTT GCT GTA GAG GGA ATT GGC TTT TC	384	0.5
23F-r	CAC AAC ACC TAA CAC TCG ATG GCT ATA TGA TTC		0.5
*cps*A-f	GCA GTA CAG CAG TTT GTT GGA CTG ACC	160	0.1
*cps*A-r	GAA TAT TTT CAT TAT CAG TCC CAG TC		0.1

Table S2. Distribution of serotypes in different specimens [% (n)]

serotypes	Invasive isolates			Non-invasive isolates		Total
	blood	cerebrospinal fluid	pleural fluid	sputum	secretions	
19F	6.4(6)	5.3(5)	5.3(5)	18.1(17)	7.4(7)	42.6(40)
19A	0	1.1(1)	0	6.4(6)	1.1(1)	8.5(8)
3	1.1(1)	0	0	7.4(7)	0	8.5(8)
6B	1.1(1)	0	0	6.4(6)	0	7.4(7)
23F	0	0	0	2.1(2)	2.1(2)	4.3(4)
18C	0	0	0	2.1(2)	0	2.1(2)
9	1.1(1)	0	0	0	1.1(1)	2.1(2)
4	1.1(1)	0	0	1.1(1)	0	2.1(2)
5	0	0	0	1.1(1)	0	1.1(1)
1	0	0	1.1(1)	0	0	1.1(1)
15	0	0	0	1.1(1)	1.1(1)	2.1(2)
16F	0	0	0	1.1(1)	0	1.1(1)
33	0	0	0	1.1(1)	0	1.1(1)
17	0	0	0	1.1(1)	0	1.1(1)
untyped	0	0	1.1(1)	11.7(11)	2.1(2)	14.9(14)
Total	10.6(10)	6.4(6)	7.4(7)	60.6(57)	14.9(14)	100.0(94)

Table S3. Distribution of different serotypes in different age groups (n)

Age groups (years)	19F	19A	3	6B	23F	15	18C	4	9	1	5	16F	33	17	untyped	Total	Proportion （%）
≤5	21	3	2	4	2	2	1	1	1	0	0	0	0	0	5	42	44.7
6~50	2	1	1	0	1	0	0	0	0	0	0	1	1	1	0	8	8.5
≥51	17	4	5	3	1	0	1	1	1	1	1	0	0	0	9	44	46.8
Total	40	8	8	7	4	2	2	2	2	1	1	1	1	1	14	94	100.0

Table S4. The serotypes distribution and coverage rates of vaccines of the 94 isolates of S.pneumoniae

serotypes	bacterial strain	Percentage (%)
19F	40	42.6
19A	8	8.5
3	8	8.5
6B	7	7.4
23F	4	4.3
15	2	2.1
18C	2	2.1
1	2	2.1
4	2	2.1
16F	1	1.0
9	1	1.0
5	1	1.0
33	1	1.0
17	1	1.0
untyped	14	14.9
The coverage rates of vaccines		
PCV7	56	59.6
PCV10	59	62.6
PCV13	75	79.6

Table S5. The serotype distribution and coverage rates of vaccines of S.pneumoniae isolates from children and elderly patients [%(n)]

serotypes	age≤5 years (n=42)	age≥51 years (n=44)	p-Value
19F	50.0(21)	38.6(17)	0.289
6B	9.5(4)	6.8(3)	0.646
23F	4.8(2)	2.3(1)	0.529
18C	2.4(1)	2.3(1)	0.973
4	2.4(1)	2.3(1)	0.326
9	2.4(1)	2.3(1)	0.326
1	0	2.3(1)	0.326
5	0	2.3(1)	-
19A	7.1(3)	9.1(4)	0.741
3	4.8(2)	11.4(5)	0.263
15	4.8(2)	0	0.143
16F	0	0	-
33	0	0	-
17	0	0	0.529
untyped	11.9(5)	20.5(9)	0.283
The coverage rates of vaccines			
PCV7	71.4(30)	54.9(24)	0.171
PCV10	71.4(30)	59.1(26)	0.233
PCV13	83.3(35)	79.5(35)	0.524

Table S6. The antibiotic susceptibility of 94 strains of S.pneumoniae（%）

Antibiotics	Resistance	Intermediate	Susceptible
Tetracycline	91.2	1.1	7.7
Erythromycin	80.2	1.1	18.7
Trimethoprim/sulfamethoxazole	63.8	13.8	22.3
Penicillin	47.3	23.1	29.7
Amoxicillin	34.1	9.9	56.0
Ceftriaxone	19.8	3.3	76.9
Cefotaxime	18.7	8.8	72.5
Meropenem	15.4	41.8	42.9
Chloramphenicol	9.9	0	90.1
Ertapenem	0	4.4	95.6
Levofloxacin	0	0	100
Moxifloxacin	0	0	100
Ofloxacin	0	1.1	98.9
Linezolid	0	0	100
vancomycin	0	0	100
